# Longitudinal Associations Between Humor Styles and Psychosocial Adjustment in Adolescence

**DOI:** 10.5964/ejop.v12i3.1065

**Published:** 2016-08-19

**Authors:** Claire Louise Fox, Simon Christopher Hunter, Siân Emily Jones

**Affiliations:** a School of Psychology, Keele University, Keele, United Kingdom; b School of Psychological Sciences and Health, University of Strathclyde, Glasgow, United Kingdom; c University of Western Australia, Perth, Australia; d Department of Psychology, Goldsmiths College, University of London, London, United Kingdom; Department of Psychology, University of Western Ontario, London, Canada

**Keywords:** humor, psychosocial adjustment, depression, loneliness, self-esteem, adolescence

## Abstract

This study assessed the concurrent and prospective associations between psychosocial adjustment and four humor styles, two of which are adaptive (affiliative, self-enhancing) and two maladaptive (aggressive, self-defeating). Participants were 1,234 adolescents (52% female) aged 11-13 years, drawn from six secondary schools in England. Self-reports of psychosocial adjustment (loneliness, depressive symptomatology, and self-esteem) and humor styles were collected at two time points (fall and summer). In cross-lagged panel analyses, self-defeating humor was associated with an increase in both depressive symptoms and loneliness, and with a decrease in self-esteem. In addition, depressive symptoms predicted an increase in the use of self-defeating humor over time, indicating that these may represent a problematic spiral of thoughts and behaviors. Self-esteem was associated with an increase in the use of affiliative humor over the school year but not vice-versa. These results inform our understanding of the ways in which humor is associated with psychosocial adjustment in adolescence.

The relationship between specific humor styles and psychosocial adjustment is poorly understood, particularly amongst children and young people. It is recognised that there are *four* main types of humor style, and that these reflect the use of humor in everyday life (Fox, Dean, & Lyford, 2013; Martin, Puhlik-Doris, Larsen, Gray, & Weir, 2003). *Self-enhancing humor* is the ability to maintain a humorous perspective in the face of stress and adversity; it is closely aligned to coping humor (e.g. ‘My humorous outlook on life keeps me from getting too upset or depressed about things’). *Aggressive humor* also enhances the self, at least in the short-term, but is done at the expense of others (e.g. ‘If someone makes a mistake I often tease them about it’). *Affiliative humor* enhances one’s relationships with others and reduces interpersonal tensions (e.g. ‘I enjoy making people laugh’). Finally, *self-defeating humor*, largely untapped by previous humor scales, is used to enhances one’s relationships with others, but at the expense of the self (e.g. ‘I often try to make people like or accept me more by saying something funny about my own weaknesses, blunders and faults’). Although individuals who use self-defeating humor might be seen as fairly ‘witty’ and ‘amusing’ (‘class clowns’), it is thought to reflect an underlying emotional neediness and low self-esteem (Martin et al., 2003).

The ability to distinguish between different components of humor has brought with it a clearer picture of the relationships between humor and adjustment. This is evidenced by the stronger correlations between humor and psychosocial adjustment which are reported when using the adult Humor Styles Questionnaire (HSQ) as compared to research using unidimensional measures (Martin et al., 2003; Gillham et al., 2011). Martin (2007) explains that there are different ways in which positive styles of humor may lead to better mental health. Firstly, humor boosts positive emotions and counteracts negative feelings of depression and anxiety. Secondly, humor can be seen as a mechanism for coping with stressful events; it can provide the individual with a way of reappraising a situation, viewing it from a different and less threatening point of view. Finally, adaptive styles of humor can lead to satisfying personal relationships, which are themselves linked to better mental health.

Among adults, affiliative and self-enhancing humor are negatively correlated with depression and loneliness, and positively correlated with self-esteem. An opposite pattern of results is found for self-defeating humor (Çeçen, 2007; Dyck & Holtzman, 2013; Fitts, Sebby, & Zlokovich, 2009; Hampes, 2005; Kuiper, Grimshaw, Leite, & Kirsh, 2004; Martin et al., 2003; McCosker & Moran, 2012; Tucker et al., 2013). In addition, aggressive humor is typically not associated with psychological adjustment but is strongly negatively correlated with social adjustment measures (Kuiper et al., 2004). For example, Kuiper et al. (2004) found that aggressive humor was negatively correlated with perceptions of interpersonal skills that included the degree of emotional support one can provide and conflict management skills. Dyck and Holtzman (2013) noted that of all the four humor styles, aggressive humor has the weakest and most inconsistent findings, with many studies that have used the adult HSQ failing to find significant associations between aggressive humor and measures of psychological adjustment.

In adolescents, the two adaptive styles of humor were negatively correlated with depression, while there was a positive correlation between self-defeating humor and depression (Erickson & Feldstein, 2007). However, the administration of the adult HSQ in this study found low reliability coefficients, particularly for the two maladaptive sub-scales (Erickson & Feldstein, 2007). The adult HSQ was subsequently adapted for use with children aged 11 years and upwards (Fox et al., 2013). With the child-HSQ, self-defeating humor was positively correlated with depressive symptoms and negatively correlated with global self-worth (Fox et al., 2013). Aggressive humor was found to be associated with the measures of psychosocial adjustment but there were some important gender differences. For example, for girls, it was linked with higher depression and lower global self-worth. Unfortunately, the cross-sectional nature of this study means that it is not possible to disentangle the causal direction of effects. It could be that particular styles of humor (e.g. adaptive) lead to improved psychosocial adjustment or that better adjustment promotes the greater use of adaptive styles of humor. Longitudinal research is therefore needed to disentangle the causal pathways between humor and psychosocial adjustment.

Research with both children and adults suggests that males and females use humor in different ways. For example, males are more likely to use aggressive humor compared to females (Fox et al., 2013; Führ, 2002; Martin et al., 2003; Saraglou & Scariot, 2002). Thus, potential gender differences in the associations between humor and adjustment were explored.

The main aim of this research was to investigate the relationships between humor styles, depressive symptoms, loneliness and self-esteem using a cross-lagged panel design. This enabled us to make stronger statements about cause and effect by predicting change across time while controlling for earlier levels of each construct. We predicted that: (a) affiliative and self-enhancing humor would be negatively associated with depressive symptoms and loneliness over time, (b) self-defeating and aggressive humor would be positively associated with depressive symptoms and loneliness over time, (c) affiliative and self-enhancing humor would be positively associated with self-esteem over time, and (d) self-defeating and aggressive humor would be negatively associated with self-esteem over time. Gender differences were also examined.

## Method

### Participants

We recruited 1,234 pupils aged 11-13 years (school years 7 and 8; 680 aged 11-12 years, and 554 aged 12-13 years), from six state secondary schools in the Midlands, UK. In terms of gender, 599 participants were male and 620 female (with missing data for 15 participants). The mean age of the sample at Time 1 was 11.68 years (*SD* = 0.64). The ethnic composition of each school (*M* = 93% white) was a reflection of the region in which the research was located; the sampling strategy took into account both rural/urban and SES profile to achieve a range of schools representative of the area from which they were recruited. Parents or carers of all pupils in the relevant year group at each school were invited to allow their child to participate, using the opt-out method of consent. Pupils who did not participate in the first session of data collection at Time 1 were not permitted to take part in the second session of data collection at Time 1; due to the number of measures administered there were two sessions of data collection at each time point. Across the time points of the study, the participation rate ranged from 70% to 85% of eligible young people registered in the schools.

Participant recruitment and data collection were conducted during school hours. Participants assented to take part in the study during class time. Classes varied in size from 10-31 with a modal class size of 24 pupils. Participants who were not taking part completed an alternative activity.

### Materials

Students completed an answer booklet at each session in which they record their name, age, school class, gender and ethnicity, prior to completion of the measures pertinent to that session.

#### Humor Styles

Participants completed the self-report child Humor Styles Questionnaire (child HSQ; Fox et al., 2013), which is an adapted version of the adult HSQ (Martin et al., 2003). Using a 4-point response scale (1 = *strongly disagree* to 4 = *strongly agree*), participants rated their agreement with the 24 statements. There are six items per sub-scale with four sub-scales in total: Self-Defeating (e.g. ‘I often put myself down when I am making jokes or trying to be funny’), Aggressive (e.g. ‘When I tell jokes I’m not worried if it will upset other people’), Affiliative (e.g. ‘I don’t have to try very hard to make people laugh – I seem to be a naturally funny person’) and Self-Enhancing (e.g. ‘I find that laughing and joking are good ways to cope with problems’). When used with 11-16 year olds, Fox et al. (2013) found acceptable levels of internal reliability for all four sub-scales (all *α* > .70), and confirmatory factor analysis identified a very clear four-factor structure. The child HSQ also has acceptable levels of test re-test reliability (*r*s range from .65 to .75 across one week). For the present study, reliability coefficients were all above .70, apart from aggressive humor at Time 1 (Time 1: *α*_aggressive_ = .66; *α*_self-defeating_ = .73; *α*_self-enhancing_ = .75, *α*_affiliative_ = .85; Time 2: *α*_aggressive_ = .71; *α*_self-defeating_ = .81; *α*_self-enhancing_ = .82, *α*_affiliative_ = .88). Mean scores were calculated for each sub-scale, with higher scores reflecting greater use of that form of humor.

#### Depressive Symptoms

The 10-item, self-report Children’s Depression Inventory - Short Form (Kovacs & Beck, 1977) for ages 7-17 years was administered. For each symptom, participants are required to indicate which of three items best describes them over the preceding two weeks, and responses were scored from 0 (no symptom), 1 (mild symptom), or 2 (moderate/severe symptom). An example item is: “I am sad once in a while”, “I am sad many times”, and “I am sad all the time.” Sum scores were then calculated, with higher scores reflecting the presence of greater symptomatology. This measure showed acceptable internal consistency with the current sample (α = .86, and α = .88, at T1 and T2 respectively).

#### Loneliness

This was assessed using the four-item, self-report Loneliness and Social Satisfaction scale (Asher, Hymel, & Renshaw, 1984; Rotenberg, Boulton, & Fox, 2005). A 5-point Likert scale ranging from 1 = *not at all true* to 5 = *really true* was used and a mean score was calculated such that higher scores reflected greater loneliness. An example item is ‘I am lonely.’ In the current study, this measure showed acceptable internal consistency (α = .86, and α = .88, at T1 and T2 respectively).

#### Self-Esteem

Rosenberg’s (1965) 10-item, self-report self-esteem measure for adolescents and adults was used with participants judging each item on a 4-point scale from 1 = *strongly disagree* to 4 = *strongly agree*. An example item is “I am able to do things as well as most people”. With the current sample, reliability coefficients were α = .87 and α = .89 at T1 and T2 respectively. Sum scores were calculated with higher scores reflecting better self-esteem.

### Procedure

Prior to data collection, the study was approved by the relevant University Ethics Committee. Data collection took place in the Fall (Time 1) and Summer (Time 2) terms of the school year, in school classrooms with a class teacher present. The average number of days between Time 1 and Time 2 was 197.5 (*SD* = 18.68). Data collection took approximately half an hour. A range of other variables were measured but are not the central focus of this paper (see Fox, Hunter, & Jones, 2015, for a paper which draws on the same dataset). Sessions began with the researchers introducing themselves and explaining the measures that would be collected that day, and explaining the confidential nature of the questionnaires. Pupils were asked to complete the questionnaire booklets in silence; they were asked to keep their answers private and not look at what other pupils were doing. Following data collection pupils were thanked and fully debriefed as to the aims and purpose of the study.

## Results

### Descriptive Statistics and Gender Differences

Descriptive statistics for the total sample and for males and females separately are shown in Table 1. A series of *t*-tests found that boys reported using more aggressive humor than females at both Time 1 and Time 2. In addition, compared to girls, boys reported higher self-esteem at both time points and fewer depressive symptoms at Time 2.

**Table 1:** Means (and SDs) for the Total Sample and Males and Females

Variables	Total sample		Male	Female	
	*M (SD)*	*N*	*M (SD)*	*M (SD)*	*t (df)*
1. T1 Agg	2.10 (0.49)	1184	2.20 (0.50)	2.00 (0.47)	7.03 (1167)***
2. T2 Agg	2.15 (0.49)	990	2.24 (0.49)	2.07 (0.48)	5.49 (978)***
3. T1 SEn	2.65 (0.53)	1187	2.68 (0.55)	2.63 (0.51)	1.67 (1170)
4. T2 SEn	2.62 (0.57)	987	2.63 (0.61)	2.62 (0.55)	0.32 (975)
5. T1 SD	2.06 (0.54)	1173	2.09 (0.55)	2.03 (0.52)	1.79 (1156)
6. T2 SD	1.95 (0.58)	990	1.97 (0.60)	1.93 (0.57)	1.08 (979)
7. T1 Aff	2.94 (0.57)	1188	2.96 (0.60)	2.92 (0.53)	1.15 (1171)
8. T2 Aff	2.96 (0.57)	991	2.98 (0.63)	2.95 (0.52)	0.72 (979)
9. T1 SEs	29.00 (5.39)	1146	29.41 (5.61)	28.59 (5.19)	2.56 (1129)**
10. T2 SEs	29.68 (5.55)	958	30.58 (5.69)	28.84 (5.32)	4.88 (946)***
11. T1 Dep	3.16 (3.54)	1103	3.09 (3.72)	3.25 (3.37)	0.77 (1088)
12. T2 Dep	3.11 (3.68)	937	2.77 (3.69)	3.45 (3.62)	2.83 (925)**
13. T1 Lone	1.78 (0.91)	1215	1.75 (0.95)	1.81 (0.88)	1.08 (1199)
14. T2 Lone	1.69 (0.85)	1015	1.65 (0.85)	1.73 (0.85)	1.50 (1003)

*Note. *SD = Self-defeating humor. Ag = Aggressive humor. Aff = Affiliative humor. SEn = Self enhancing humor. Dep = Depressive symptoms. SEs = Self-esteem. Lone = Loneliness. T1 = Time 1. T2 = Time 2.

*p < .05. **p < .01. ***p < .001.

### Correlations

As shown in Table 2, all four humor styles and all three measures of adjustment showed moderate to high stability (*r*s ranging from .50 to .63). At Time 1 and Time 2, self-enhancing and affiliative humor were positively correlated with self-esteem and negatively correlated with depressive symptoms and loneliness. Self-defeating humor, in contrast, was negatively correlated with self-esteem and positively correlated with depressive symptoms and loneliness. The same patterns were observed from Time 1 to Time 2. For example, self-defeating humor at Time 1 was positively correlated with depressive symptoms at Time 2, and depressive symptoms at Time 1 were positively correlated with self-defeating humor at Time 2.

**Table 2:** Intercorrelations for Psychosocial Adjustment and Humor Styles at Time 1 and Time 2

Variables	Correlations													
	1.	2.	3.	4.	5.	6.	7.	8.	9.	10.	11.	12.	13.	14.
1. T1 Agg	--													
2. T2 Agg	.50***	--												
3. T1 SEn	.03	.02	--											
4. T2 SEn	-.01	.01	.51***	--										
5. T1 SD	.14***	.15**	.09**	-.05	--									
6. T2 SD	.07*	.20***	.04	.02	.55***	--								
7. T1 Aff	.13***	.13***	.36***	.21***	-.16***	-.19***	--							
8. T2 Aff	.11**	.14***	.22***	.29***	-.15**	-.29***	.63***	--						
9. T1 SEs	-.06*	-.04*	.23***	.17***	-.42***	-.37***	.32***	.31***	--					
10. T2 SEs	-.01	-.06*	.11***	.20***	-.36***	-.54***	.24***	.35***	.61***	--				
11. T1 Dep	-.02	.00	-.23***	-.21***	.40***	.40***	-.31***	-.29***	-.71***	-.52***	--			
12. T2 Dep	.04	.01	-.13***	-.29***	.35***	.54***	-.25***	-.36***	-.52***	-.77***	.59***	--		
13. T1 Lone	-.07*	.03	-.15***	-.16***	.39***	.37***	-.33***	-.31***	-.61***	-.44***	.72***	.56***	--	
14. T2 Lone	-.07*	-.04	-.12***	-.22***	.34***	.53***	-.28***	-.37***	-.44***	-.67***	.51***	.78***	.54***	--

*Note.* SD = Self-defeating humor. Ag = Aggressive humor. Aff = Affiliative humor. SEn = Self enhancing humor. Dep = Depressive symptoms. SEs = Self-esteem. Lone = Loneliness. T1 = Time 1. T2 = Time 2.

*p < .05. **p < .01. ***p < .001.

At Time 1 and Time 2 aggressive humor was negatively correlated with self-esteem. Also at Time 1 aggressive humor was negatively correlated with loneliness. Aggressive humor at Time 1 was negatively correlated with loneliness at Time 2 and self-esteem at Time 1 was negatively correlated with aggressive humor at Time 2. However, these correlations for aggressive humor were very small, ranging from -.04 to -.07 (see Table 2).

### Structural Equation Modelling

#### Measurement Models

AMOS 20.1 was used to estimate a fully cross-lagged model evaluating the proposed relationships between the three psychological adjustment variables and humor styles. Data from only those participants who took part at Time 1 (*n* = 1,234) were included in the analyses, and Full Information Maximum Likelihood was used in the analyses to deal with missing data. Because *χ*^2^ values are inflated by large sample sizes, we used additional criteria to assess model fit, including the CMIN/DF ratio, CFI and RMSEA. A good fitting model is indicated by CMIN/DF values under 3 (Hu & Bentler, 1999); CFI above .90, reflecting adequate fit (Bentler, 1992), and above .95 to indicate good fit; and RMSEA scores of .06 or less (Hu & Bentler, 1999). First, separate measurement models were assessed for: 1) loneliness, 2) depressive symptoms, and 3) self-esteem, using the T1 and T2 data. In each case a path was drawn with the T1 latent variable predicting the T2 latent variable. T1 and T2 error variances for identical observed variables were permitted to covary. Model parameters were examined and in parallel we also considered whether there were any theoretical justifications for the modifications. For loneliness, the fit of the model was good (see Table 2). For depressive symptoms and self-esteem the goodness of fit statistics indicated that modifications would be needed (e.g. CMIN/DF scores of 5-6). For depressive symptoms, we tested a second model where we allowed focussed sets of error terms to covary. These were error terms for specific items which reflected methodological rather than conceptual issues i.e. negatively worded items. This provided a better fitting model (see Table 3 for the comparison of Models 2 and 3). In the same way, for self-esteem, we allowed the error terms for the five negatively worded items to covary. Again, this led to a better fitting model (see Table 3 for the comparison of Models 4 and 5). Stability paths from T1 to T2 were: .63 for loneliness, .68 for depressive symptoms and .67 for self-esteem.

**Table 3:** Cross-Lagged Models

Model	*χ*^2^	*df*	*χ^2^/df*	CFI	RMSEA [90% CI, LOW, HIGH]
Model 1: Loneliness	51.12***	16	3.20	.99	.042 [.030, .055]
Model 2: Depressive symptoms	908.76***	162	5.61	.91	.061 [.057, .065]
Model 3: Model 2 + constraints	643.49***	140	4.60	.94	.054 [.050, .058]
Model 4: Self esteem	940.11***	159	5.91	.92	.063 [.059, .067]
Model 5: Model 4 + constraints	444.76***	139	3.20	.97	.042 [.033, .047]
Model 6: Humor styles	2515.22**	1029	2.44	.91	.034 [.033, .036]
Model 7: Model 6 + constraints	2350.82***	1023	2.30	.92	.032 [.031, .034]
Model 8: Psychosocial adjustment and humor styles	9094.0***	4280	2.13	.90	.030 [.029, .031]

***p < .001.

For the humor styles measurement model, T1 latent variables predicted all T2 latent variables. Since humor was multidimensional, all the T1 latent humor style variables were allowed to covary, as were the T2 latent variable disturbances. Corresponding T1 and T2 error variances for the observed variables were also permitted to covary. The predicted four factor model provided an adequate fit. The error terms for the three negatively worded aggressive humor questionnaire items were allowed to covary, and this provided a better fitting model (see Table 3 for a comparison of Models 6 and 7). Stability paths from T1 to T2 ranged from *β* = .62 to .70.

#### Cross-Lagged Structural Equation Models

**Figure 1:**
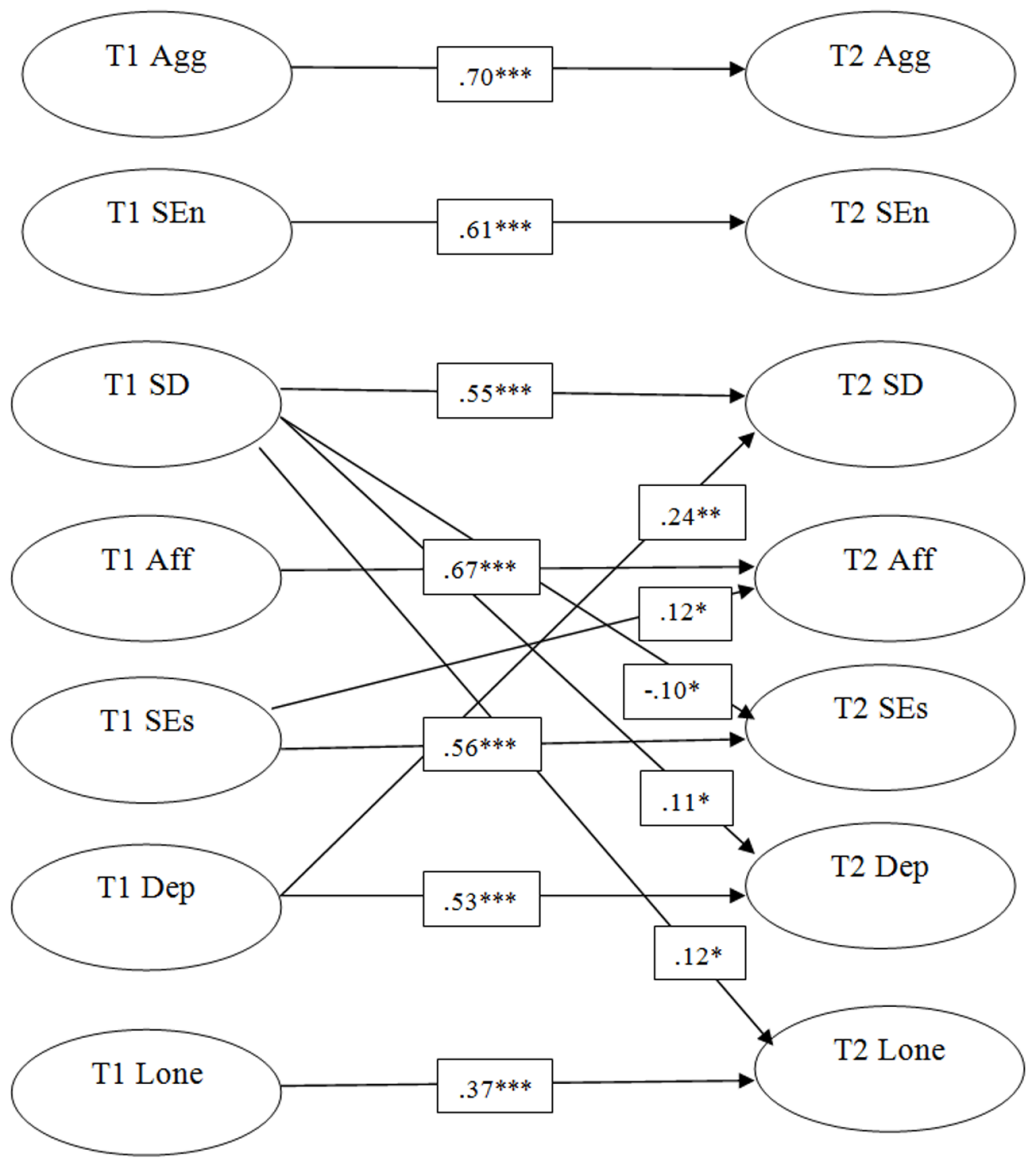
Schematic of structural model for psychosocial adjustment and humor styles (omitting error terms, indicators for latent variables and covariance paths). *Note.* Only significant paths shown. Stability coefficients appear in the middle of the diagram. *p < .05. **p < .01.

Having established the adequacy of the measurement models, a cross-lagged model combining all three psychosocial adjustment variables and self-reports of humor styles was evaluated. All the T1 latent variables were allowed to covary, as were the T2 latent variable disturbances. This model (see Table 3, Model 8) displayed an acceptable fit. See Figure 1 for a schematic depiction of the model with all significant paths. Self-defeating humor at Time 1 predicted an increase in depressive symptoms and loneliness at T2 and a decrease in self-esteem (T2). There was a reciprocal relationship between self-defeating humor and depressive symptoms to the extent that levels of each at T1 predicted increases in the other at T2. In addition, there was a significant path between self-esteem at T1 and affiliative humor at T2.

#### Multiple Groups Analyses

We conducted analyses to assess whether model parameters were equivalent for males and females. To achieve this, we compared two models. The first model was an unconstrained model in which all stability and cross-lagged paths were allowed to vary across boys and girls. The second model constrained all stability and cross-lagged paths to be equal across boys and girls. If the relative fit of the constrained model is significantly worse than the unconstrained model, using change in chi-square (∆*χ^2^*) as an indicator, then we can conclude that effects differ across groups. There was no significant decrement in fit for the constrained versus the basic model (∆*χ^2^* = 43.92, *df* = 49, *p* > .05). This indicates that the stability and cross-lagged paths are equivalent for boys and girls.

## Discussion

This is the first study to examine the longitudinal associations between children’s humor styles and their psychosocial adjustment. As noted by Martin (2007), there is a growing body of evidence to suggest that humor in adults is linked to better mental and physical health, but there is a lack of research on the social and emotional benefits of humor in children. We found that self-defeating humor predicted an increase in loneliness over time and a decrease in self-esteem. There was evidence of a bi-directional relationship with self-defeating humor predicting an increase in depressive symptoms over time *and* vice-versa. In addition, self-esteem predicted an increase in the use of affiliative humor. Contrary to our expectations, these relationships were the same for boys and girls.

Despite the large number of studies over the last decade which have used the adult HSQ, we are not aware of any studies which have examined the longitudinal relationships between the four humor styles and aspects of psychosocial adjustment. We found that having a high self-esteem can facilitate the development of affiliative humor over time and that depressive symptoms can lead to a greater use of self-defeating humor. Future studies could examine potential mediating variables that might explain these relationships. In particular, the role of self-defeating humor in the development of depressive symptoms should be examined further. Our findings suggest that adolescents may get caught up in a vicious cycle when using self-defeating humor, with one problem exacerbating the other; how a child feels about the self and others may be reflected in their humor, which in turn, may reinforce negative self-referential cognitions, leading to an increase in depressive symptoms. Support for such a proposition comes from theories of depression, e.g. Beck and colleagues, who argued that negative core beliefs about the self can contribute to depression (Beck, Rush, Shaw, & Emery, 1979; Dozois & Beck, 2008). Furthermore, it has been found that individuals with maladaptive self-schemas are more likely to display maladaptive coping (Young et al., 2003). Self-defeating humor could be viewed as a type of maladaptive coping, which might reinforce and maintain these beliefs, leading to an increase in depression. It may have the same effect as rumination, often linked with depression, which is thought to bring negative thoughts to the ruminator’s attention, facilitating a negative interpretation of their situation (Lyubomirsky & Nolen-Hoeksema, 1995; Nolen-Hoeksema, 2000).

Support for the role of negative self-beliefs comes from cross-sectional research in adult populations where humor styles mediated the relationships between early maladaptive schemas and depressive symptoms (Dozois, Martin, & Bieling, 2009). The authors suggest that humor, employed as a coping strategy, could be a useful avenue for intervention, particularly in relation to depression. The therapist and child could collaboratively discuss how they use humor, establishing whether this leads to unintended negative consequences. Cognitive Therapy draws on the links between beliefs and behaviour, and how this can lead to distress. If the child can recognise that their use of humor does have some drawbacks that they were not aware of, attention could be paid to developing more adaptive styles of humor to see what impact that has on the child’s psychological state.

As well as self-defeating humor reinforcing beliefs about the self, this style of humor also involves one’s weaknesses being validated by others (e.g. through others’ laughter), which can leave an individual feeling unsupported and that close family and friends do not care. This may compound the negative effects of self-defeating humor. In support of this, social support has been identified as an important mediator of the relationship between humor styles and well-being. Dyck and Holtzman (2013) found that the association between self-defeating humor and depressive symptoms in adults was mediated by (lack of) social support.

An additional cautionary note is that the means for depressive symptoms are fairly low given that the potential range of scores is from 0 to 20. However, the scores showed expected and significant correlations with other variables measured as part of the study, suggesting that there is sufficient variation in the sample to demonstrate the relationships we predicted. For example, depression was associated with all but one of the humor styles. However, it is possible that within our sample there were very few young people who were displaying clinically significant levels of depressive symptoms. Further research could endeavour to explore the associations with more ‘at risk’ populations.

It was also found that self-defeating humor led to an increase in loneliness across time. This finding compliments our findings reported elsewhere which suggested that use of this humor style may put adolescents at an increased risk of peer victimisation over time (Fox et al., 2015). It has been argued that humor is one means through which social status can be achieved and maintained (Klein & Kuiper, 2006). The humor style a child used may impact on their social relationships because different humor styles are more positively received by others (see Kuiper, Kirsh, & Leite, 2010; Zeigler-Hill et al., 2013). If humor does impact on children’s peer groups relationships, then this can potentially lead to greater feelings of loneliness. This effect may also be mediated by social support, as indicated above. Future studies could measure the role of perceived social support when examining the negative effects of self-defeating humor.

Somewhat surprisingly, there was no evidence for a protective role for humor in the development of adjustment problems. Affiliative humor did not predict changes in adjustment over time, although self-esteem did predict an increase in the use of affiliative humor. Self-enhancing humor was unrelated to the measures of psychosocial adjustment across time. This casts some doubt on the validity of this measure but it may simply indicate that it is only in adulthood that we can use this style of humor effectively to cope with life stress and adversity (Altshuler & Ruble, 1989). In support of this point, interviews with a small sample of 12-16 year olds elicited open-ended responses to suggest that children of this age do see a use for humor as a coping tool, but only with less serious events (Führ, 2001), e.g. ‘does not always help, not with serious things’. This null finding only emerged within the full causal model, which, it could be argued, is a more conservative test of the associations between multiple predictors and several different outcome variables, especially since this controlled for earlier levels of each outcome variable. Indeed, even those paths that were significant were small in magnitude. Simple correlations identified cross-sectional *and* longitudinal associations between self-enhancing humor and all three measures of psychosocial adjustment. Future research should examine the role of this particular humor style further to advance understanding of the development of self-enhancing humor as a coping tool across adolescence.

The same relationships were identified for boys and girls, in contrast to our previous cross-sectional research which identified gender differences in the associations between aggressive humor and adjustment (Fox et al., 2013). In our previous study, aggressive humor was associated with higher depression and lower global self-worth in girls. In the current study, there were no longitudinal associations between aggressive humor and all three of the measures of psychosocial adjustment. As indicated by Dyck and Holtzman (2013), of all the four humor styles, aggressive humor demonstrates the weakest and most inconsistent findings, with many studies using the adult HSQ failing to find significant associations between aggressive humor and measures of psychological adjustment. Our findings add to the growing body of literature which suggests that aggressive humor is unrelated to measures of psychological adjustment, at least in the short-term. As argued by Martin (2007), over the long-term, aggressive humor may be detrimental to the self because it tends to alienate others. Studies over a longer time-frame may be able to identify significant associations between aggressive humor and psychological maladjustment.

Our study employed a cross-lagged panel-design, allowing us to begin to describe the direction of possible effects between variables. However, our study is not without limitations. One such limitation is the use of self-reports to measure humor styles and psychosocial adjustment, which raises the possibility that associations were biased by shared method variance. However, it is not clear that shared method variance necessarily leads to inflated estimates (Conway & Lance, 2010). Regardless, it would be judicious for future research to consider gathering data from different sources, such as peers, teachers or parents.

In sum, this if the first study to identify longitudinal associations between humor styles and psychosocial adjustment, and in children. There is little need for further cross-sectional studies and we would encourage researchers to move on to address the causal direction of effects in adults and children, so that we can advance understanding of the development of humor and its negative consequences. A bi-directional relationship was identified between self-defeating humor and depressive symptoms. A consideration of other potential moderating and mediating variables such as negative self-beliefs and perceived social support would be useful avenues for future research. The findings suggest that a focus on humor use in the treatment of childhood depression could be beneficial.
